# Why Variation in Flower Color May Help Reproductive Success in the Endangered Australian Orchid **Caladenia fulva**

**DOI:** 10.3389/fpls.2021.599874

**Published:** 2021-02-09

**Authors:** Georgia Basist, Adrian G. Dyer, Jair E. Garcia, Ruth E. Raleigh, Ann C. Lawrie

**Affiliations:** ^1^School of Science, RMIT University, Bundoora, VIC, Australia; ^2^Bio-inspired Digital Sensing Lab, School of Media and Communication, RMIT University, Melbourne, VIC, Australia; ^3^Department of Physiology, Monash University, Melbourne, VIC, Australia; ^4^Royal Botanic Gardens Melbourne, South Yarra, VIC, Australia

**Keywords:** orchid, endangered, flower color, pollination, Hymenoptera, fruit set, DNA analysis

## Abstract

*Caladenia fulva* G.W. Carr (Tawny Spider-orchid) is a terrestrial Australian endangered orchid confined to contiguous reserves in open woodland in Victoria, Australia. Natural recruitment is poor and no confirmed pollinator has been observed in the last 30 years. Polymorphic variation in flower color complicates plans for artificial pollination, seed collection and *ex situ* propagation for augmentation or re-introduction. DNA sequencing showed that there was no distinction among color variants in the nuclear ribosomal internal transcribed spacer (ITS) region and the chloroplast trnT-trnF and matK regions. Also, authentic specimens of both *C. fulva* and *Caladenia reticulata* from the reserves clustered along with these variants, suggesting free interbreeding. Artificial cross-pollination *in situ* and assessment of seed viability further suggested that no fertility barriers existed among color variants. Natural fruit set was 15% of the population and was proportional to numbers of the different flower colors but varied with orchid patch within the population. Color modeling on spectral data suggested that a hymenopteran pollinator could discriminate visually among color variants. The similarity in fruiting success, however, suggests that flower color polymorphism may avoid pollinator habituation to specific non-rewarding flower colors. The retention of large brightly colored flowers suggests that *C. fulva* has maintained attractiveness to foraging insects rather than evolving to match a scarce unreliable hymenopteran sexual pollinator. These results suggest that *C. fulva* should be recognized as encompassing plants with these multiple flower colors, and artificial pollination should use all variants to conserve the biodiversity of the extant population.

## Introduction

Conservation of endangered species is a high priority for members of the International Union for Conservation of Nature.^1^ Such governments around the world are obligated to devise and fund strategies to prevent the extinction of endangered species, in order to conserve biodiversity. The Orchidaceae is one of the most threatened plant families, as it has large numbers and proportions of endangered species worldwide (Wraith and Pickering, 2019).

### *Caladenia* and Conservation

In temperate Australia, *Caladenia* species are widespread terrestrial orchids that produce a single leaf, may produce a single flower (occasionally 2), perennate by annual tubers and are active above-ground during cooler, wetter months, typically from late autumn through winter to spring (Jones, 2008). However, 18% of *Caladenia* species are threatened and protected under the Environment Protection Biodiversity and Conservation Act (EPBC) 1999 (Australian Government Department of Agriculture Water and the Environment, 2018). In the State of Victoria alone, 53% of the *Caladenia* species are listed as protected flora under the Victorian Flora and Fauna Guarantee Act (FFG) 1988 (Victorian Government Department of Environment, Land, Water and Planning, 2019). Most of these species are the very attractive spider-orchids in the subgenus Calonema (Hopper and Brown, 2004; Hopper, 2009; Clements et al., 2015). These are so named because of the long tapering tepals reminiscent of spider-legs.

**FIGURE 1:**
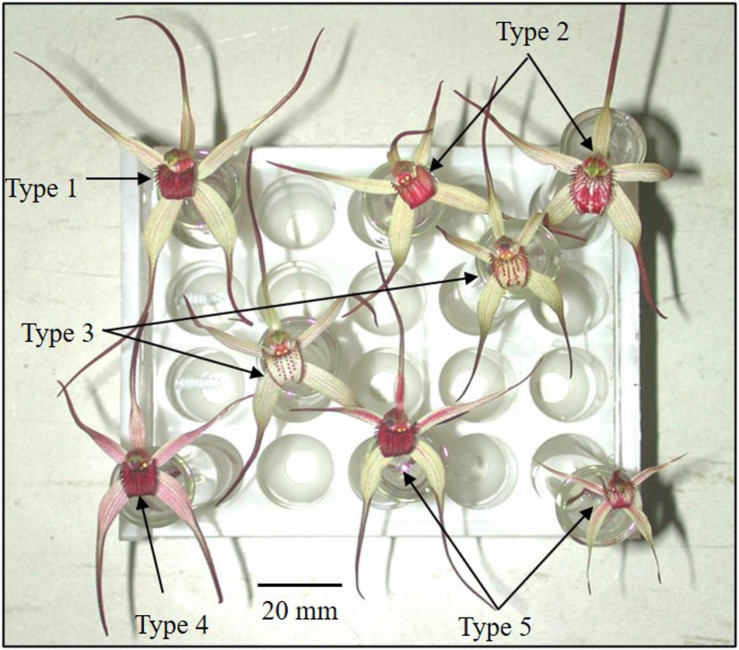
Polymorphic flower color categories of *Caladenia* species from Deep Lead imaged with white light.

One such threatened spider orchid is *Caladenia fulva* (Carr, 1991; Figure 1). The species is categorized as nationally endangered, and has had recovery plans under the EPBC and FFG for the last 18 years (Coates et al., 2002). Recovery plans for this, as for other endangered species, specify studying its biology and monitoring populations and their reproduction in the wild. Artificial pollination and seed collection are used to establish *ex situ* populations for conservation and use in possible augmentation or re-introduction *in situ* if natural pollination is low or absent (Coates et al., 2002). The major aim is to establish self-maintaining populations large enough to survive without human intervention.

In *C. fulva*, no pollinator has been found despite baiting attempts (Colin Bower, personal communication) and plans for artificial pollination for seed production have been complicated by variation in flower color. *C. fulva* was described as having tawny (fawn)-colored tepals (three sepals and two petals) and a dark crimson labellum (Carr, 1991). The numerous color variants have various degrees of red blotching and streaks of red pigmentation in the tepals and labellum (Figure 1). The polymorphy in flower color has been speculated as being due to hybridization with other spider-orchids, especially *Caladenia reticulata* Fitzg. (Backhouse and Jeanes, 1995), but there has been no critical evidence to support this claim.

The uncertain status of the color variants, their unknown reproductive rates and the lack of an identified pollinator make it difficult to decide if all variants should be included or excluded in plans for artificial pollination. If the progeny are viable, either strategy could change the genetics of the populations and hence affect the conservation of the species. Molecular methods based on sequencing of nuclear and plastid genomes have been previously used in orchids to resolve taxonomic questions (Jones et al., 2001; Hopper, 2009; Breitkopf et al., 2015; Jin et al., 2017; Scaccabarozzi et al., 2018; Baguette et al., 2020) including in *Caladenia* (Swarts et al., 2014; Clements et al., 2015). Adopting these molecular methods is vital in the recovery plan to provide such information on population structure. It is also vital to test for the feasibility of cross-pollinating among color variants to find if fruits are formed and the seeds are viable.

### *Caladenia* and Pollination

Most *Caladenia* spider-orchids investigated since the seminal paper by Stoutamire (1983) are pollinated by sexual deception of thynnine wasps (Hymenoptera-Thynnidae-Thynninae), e.g., Bohman et al. (2018). The male thynnine wasp is firstly attracted long-range (up to 10 m) by scents that imitate the pheromones of the wingless females (Bower, 1996; Schiestl, 2005; Peakall and Whitehead, 2014; Bohman et al., 2017; Phillips and Peakall, 2018; Wong et al., 2017). In such a case, color is therefore not the main or only attractant but links between flower color and odor have been demonstrated in other deceptive orchids (Dormont et al., 2014, 2019) and high contrast both within the flower and against the background is important (Gaskett et al., 2017; Phillips and Peakall, 2018).

Once such a pollinator lands on the central labellum of a sexually deceptive orchid, it attempts vigorously to copulate with the flower, thus acquiring the pollinia and depositing previously acquired pollen, before leaving to seek a mate elsewhere, typically outside the same orchid patch. For example, the thynnine wasp pollinators of the sexually deceptive *Drakaea glyptodon* and *Caladenia tentaculata* avoided multiple flowers within the same patch and in the latter case flew to a mean 17 m and up to almost 60 m away before depositing pollen (Peakall, 1990; Peakall and Beattie, 1996).

Bees and wasps (Hymenoptera) are the most common pollinators of sexually deceptive orchids (Bohman et al., 2018) and have the critical ability to learn the odor and appearance of flowers after unsuccessful attempts to mate (Baguette et al., 2020). Flower color polymorphism may be advantageous by mitigating against the pollinator learning to avoid non-rewarding flowers (Wong and Schiestl, 2002; Wong et al., 2004; Schiestl, 2005; Jersáková et al., 2006; Gaskett and Herberstein, 2010; Gaskett, 2011; Dormont et al., 2014; Paulus, 2018; Dyer et al., 2019; Baguette et al., 2020). This has been termed negative frequency-dependent selection (Schiestl, 2005) and has the effect of keeping rarer flower types in the population. This is especially important in endangered orchids, in which populations are frequently small and pollination observations are limited (Tremblay et al., 2005; Phillips et al., 2009a, 2017).

Pollinators can be shared among orchids that overlap in range and flowering period (Joffard et al., 2019), but are shared less commonly among sexually deceptive species than among food-deceptive or rewarding (nectariferous) species (Phillips and Peakall, 2018; Joffard et al., 2019). However, most species investigated within the “*Caladenia reticulata/patersonii* complexes” (to which *C. fulva* belongs) shared pollination by one thynnine wasp – *Phymatothynnus nitidus* (Swarts et al., 2014). They thus formed one potentially interbreeding population in south-eastern Australia, over an area that overlaps that of *C. fulva.* At the other extreme, pollinator specialization has been found even within morphologically uniform populations of orchids and their pollinators (Bower and Brown, 2009; Phillips and Peakall, 2018), as well as within the *P. nitidus* complex (Phillips et al., 2009a).

Flower color variation in *C. fulva* could be significant in determining relative pollination and hence reproduction rate. Therefore, any selectivity in artificial pollination could affect conservation efforts for *C. fulva*. If one of the flower color phenotypes is more successful in pollination than the others, as in *Dactylorhiza sambucina* (Gigord et al., 2001), the population would be liable naturally to drift to a greater frequency of that phenotype or to reproductive isolation, as suggested in *Ophrys* evolution in Europe (Breitkopf et al., 2015). Counting the frequency of the flower color phenotypes in the *Caladenia* population and their success in fruiting would assist the recovery plan to monitor their relative frequency and likelihood of forming the next generation. Also testing the spectral qualities of flower color variants could assist the recovery plan by predicting which color variant(s) would be most attractive to hymenopteran pollinators.

### Aims

The aims of this study were to assist the recovery plan for conservation of *C. fulva* by investigating (1) if DNA-based molecular grouping separated the flower color variants from one another and from “typical” *C. fulva* and *C. reticulata*, (2) if artificial pollination across flower color variants could produce viable seed, (3) if fruit formation varied among flower color variants after natural pollination, and (4) if a hymenopteran pollinator could discriminate amongst the different flower colors as seen by humans. Answers to these questions would benefit the recovery plan by clarifying if the flower color variants formed one interbreeding population and if artificial pollination was likely to change its structure. Finally, a rationale is proposed for the existence of polymorphic flower color in *C. fulva*.

## Materials and Methods

### Site and Plants

The plants studied were growing at Deep Lead near Stawell in Western Victoria, Australia (latitude 37.0717°S, longitude 142.7908°E). The mean annual rainfall is 473.0 mm, the mean daily maximum temperature 20.6°C and the mean daily minimum temperature 8.5°C (Australian Government Bureau of Meteorology, 2020). The vegetation comprised of open woodland with *Eucalyptus leucoxylon* F.Muell. (yellow gum) as the dominant tree and a very sparse understory of the shrubs *Acacia pycnantha* Benth., *Acrotriche serrulata* R.Br., *Grevillea alpina* Lindl., *Micromyrtus ciliata* (Sm.) Druce, various grasses, and herbaceous perennials including 42 different orchid species (Carr et al., 1989). The area was assessed in the 2005 Ecological Vegetation Class (EVC) as a mixture of No. 61 Box-Ironbark Woodland and No. 48 Heathy Woodland with a strategic biodiversity value ranking of 100 (Victorian Government Department of Environment, Land, Water and Planning, 2020). The soil was a uniform red-yellow sandy gravel with a pH of 4.4 (CaCl_2_) – 5.4 (water).

*Caladenia fulva* currently occurs only in part of two contiguous small flora and fauna reserves totaling 1120 ha in open woodland at Deep Lead, near Stawell, Victoria. Deep Lead was one of the richest alluvial fields in the Victorian goldfields and was highly disturbed during goldrushes in 1857–1878 and subsequent mining. *C. fulva* was first named in 1991 after a comprehensive study of the vegetation at Deep Lead (Carr et al., 1989; Carr, 1991). The description of *C. fulva* was based on one of the flower color variants at the site and the others were noted as being worthy of further investigation (Carr, 1991). The orchid is a summer-dormant herbaceous perennial that perennates by an annual succession of underground tubers that produce one green leaf and may produce a solitary flower each year. Its estimated population is 650 (Threatened Species Scientific Committee, 2016).

*Caladenia fulva* plants that grew in five patches 50–200 m apart were used for the study. Flowering plants were tagged and typed on 1–2 days in mid-flowering season in each of 2000 (81 plants), 2001 (49 plants), 2003 (70 plants), and 2004 (64 plants) (total of 264 plants). Each plant was only used once. The position of each orchid was tagged in the ground using a 2 mm stainless steel mini tent peg (Wiretainers, Brunswick East, Australia) to which was attached a uniquely numbered weatherproof pallid green plastic animal swivel tag (Stewart Farm Supplies).

### Categorization of Color Variants

Each flower was categorized as one of five categories (Table 1 and Figure 1) on the basis of tepal and labellum color. Category 1 corresponded to the type description of *C. fulva* (Carr, 1991; Geoff Carr, personal communication). Category 5 was closest to the type description of *C. reticulata* Fitz. A range of authentic flowers for each species is shown in a range of resources, including in the Flora of Victoria (Walsh and Entwisle, 1994, updated version online at https://vicflora.rbg.vic.gov.au/), Backhouse and Jeanes (1995), Jones (2008), Jeanes (2015), and the Atlas of Living Australia (2020).^2^

**TABLE 1:** Characteristics of polymorphic types of polymorphic *Caladenia* species at Deep Lead, and of *C. fulva* and *C. reticulata* (information on known species from Backhouse and Jeanes, 1995).

**Species or type**	**Tepal color (to human eyes)**	**Labellum color (to human eyes)**
*Caladenia fulva*	Yellow-green ± fine crimson stripes	Solid crimson
Category 1	Yellow-green ± fine crimson stripes	Solid crimson
Category 2	Yellow-green + crimson stripes or blotches	Crimson blotches or crimson with central white stripe
Category 3	Yellow-green or light yellow ± fine crimson stripes	Yellow-green or light yellow
Category 4	Pink-peach + crimson stripes	Solid crimson
Category 5	Yellow-green + thick central crimson stripe	Crimson, either solid or with blotches or central white stripe
*Caladenia reticulata*	Pale creamy yellow + basal red streaks and blotches	Solid crimson

*See text for locations of illustrations of authentic species.*

To record how closely mixed were the different categories of flower color, each tagged orchid in patches 1–4 was mapped in 2000–2001 using a theodolite relative to a tagged arbitrary point (0,0). A theodolite was used to record the distances between plants of different categories, as GPS accuracy was inadequate due to the close spacing (frequently < 5 cm) between individuals. Measurements recorded were bearing and distant from point (0,0), which was marked by a post in the ground. The mapping data were graphed manually to give (x,y) coordinates that were entered into a Microsoft Excel spreadsheet to produce a map of the tagged orchids that flowered in 2001.

### Molecular Analysis

DNA was extracted from leaf tips of 31 plants that flowered in 2001 using a Qiagen DNeasy Plant Mini kit according to the manufacturer’s instructions. Sample size was limited (4–80 mg, average of 36 mg) because the species is endangered. DNA was also extracted from five samples of each of authentic “*C. fulva*” and “*C. reticulata*” collected from Deep Lead (the latter generously gifted by I. and T. McCann of the Stawell Field Naturalists Club via Neville Walsh of the Royal Botanic Gardens Melbourne). All authentic specimens had identities confirmed by staff at the Royal Botanic Gardens before use.

#### Sequencing

One nuclear (ribosomal internal transcribed spacer – ITS) and several regions of the chloroplast genes (trnT-F, trnK) were sequenced as used previously for *Caladenia* (Hopper and Brown, 2004; Swarts et al., 2014; Clements et al., 2015). The ITS region was amplified using the universal primers ITS1 and ITS4 (White et al., 1990) as described previously (Reiter et al., 2018a). Four regions of the chloroplast genome were also sequenced using previously published primers and conditions: trnT-trnL, trnL intron, trnL-trnF (Taberlet et al., 1991) and the latter part of the matK region (here designated matK2) of the trnK intron (Sauquet et al., 2003; Steane et al., 2003). Each product was purified and sequenced as described previously (Reiter et al., 2018a). Products were electrophoresed at Micromon (Monash University).^3^

Phylogenetic trees for each type of sequence were constructed from ClustalW alignment (Thompson et al., 1994) using the Maximum Likelihood method based on the Tamura-Nei model (Tamura and Nei, 1993) with 500 bootstraps (Felsenstein, 1985) in MEGA7 (Kumar et al., 2016).^4^ Sequences for *C. fulva* have been deposited in GenBank as Accession Numbers MT894435-MT894470 (ITS), MT914511-MT914554 (trnT-trnL), MT914555-MT914601 (trnL intron), MT950637-MT950682 (trnL-trnF), and MT966280-MT966314 (matK2). For outgroups, comparable sequences for species from other sections of *Caladenia* were obtained from GenBank using NCBI (National Center for Biotechnology Information).^5^ Genetic distances (d) within and between categories were calculated using Jukes-Cantor analysis in MEGA7.

Sequences were also concatenated for all those samples (as in Swarts et al., 2014; Clements et al., 2015; Joffard et al., 2019) with all five sequences to give an overall assessment of the phylogenetic relationships among the phenotypes and authentic species. Concatenated sequences were analyzed by Maximum Likelihood analysis in MEGA7 and also by the MrBayes 3.2.6 plugin (Huelsenbeck and Ronquist, 2001) in Geneious Prime 2021.0.3^6^ using the HKY85 substitution model, a burn-in of 100,000, a subsampling frequency of 200 and a chain length of 1,100,000 with Plant 5, Category 2 as the outgroup.

#### Inter-Simple Sequence Repeats

Inter-simple sequence repeats (ISSR) was used to assess diversity among polymorphic phenotypes in other regions of their DNA using microsatellite primers (CAT)_5_, (GTG)_5_, and (GACA)_4_, similarly to the study by Swarts et al. (2014). Each 25 μL reaction contained: 12.5 μL Promega GoTaq Green Master Mix, 9.5 μL nuclease-free water, 1 μL microsatellite primer (25 μM) and 2 μL containing 5–20 ng of genomic DNA or sterile nuclease-free water. Thermocycling for each primer was as described by Elmeer et al. (2011) for (CAT)_5_ and by Ryberg et al. (2011) for (GTG)_5_ and (GACA)_4_. PCR products were separated by electrophoresis and products recorded as before. The presence or absence of each amplicon was entered into a binary matrix in the statistical program Minitab Version 18^7^ and individuals were grouped by similarity using multivariate analysis (principal components analysis and cluster analysis with complete linkage and squared Euclidean distance at *p* = 0.05). Genetic diversity was estimated by the number of expected alleles (N_*e*_), observed (H_0_), and expected (H_*e*_) heterozygosity in GenAlEx6.51 (Peakall and Smouse, 2006, 2012). AMOVA was used to estimate the degree of population genetic variation (φ_*PT*_) (equivalent of F_*ST*_ for binary data).

### Pollination Studies

#### Natural Pollination

To investigate if the different individuals varied in success in fruiting varied with color morphotype or patch, 34 of the orchids tagged in 2000–2001 were observed for natural pollination, as judged by fruit set. Since these were the more common types not needed for the pollination scheme in 2001, there was the possibility of bias and a limited number of types. Therefore, natural pollination was also monitored separately in later years by typing and tagging individual flowering orchids and noting if they produced capsules. No data were collected in 2002 because all orchids were heavily grazed and did not flower due to the ongoing drought. A total of 76 orchids flowered and were tagged in 2003, when the drought broke, and 73 in 2004. Data on differences in flowering (2000, 2003, and 2004) and fruiting (2003 and 2004) with category and patch were organized into contingency tables and analyzed by means of Chi-square tests and other measures of association (Hartl and Clarke, 2007) against a null hypothesis of no difference among categories and patches using Minitab Version 18.

#### Artificial Pollination

All available flowering plants were artificially cross-pollinated between and within color variants in each of 2000 and 2001 years according to a previously determined matrix to incorporate all possible combinations between and within the five categories (Table 2 and Supplementary Table 1): 48 plants on September 25, 2000 and 33 plants on September 12 and 24, 2001 (total of 81 plants). To avoid bias, each plant was only used in 1 year. The number of flowering orchids tagged was dependent on their frequency over both years and did not represent the five categories equally.

**TABLE 2:** Variation in flowering with category and patch in which they grew in unbiased tagged plants of *Caladenia* species at Deep Lead in 2000, 2003, and 2004.

**Criterion**	**No. flowers**				
	**2000**	**2003**	**2004**	**Total**	**%**
**Category**					
1	15	17	13	45	21.20.5
2	26	18	12	56	26.41.9
3	14	23	27	64	30.21.8
4	19	8	4	31	14.62.1
5	4	4	8	16	7.50.6
Total	78	70	64	212	100.0
**Patch**					
1	28	15	20	63	29.71.8
2	50	14	10	74	34.96.0
3	0	7	3	10	4.71.0
4	0	8	8	16	7.51.3
5	0	26	23	49	23.13.9
Total	78	70	64	212	100.0

Only flowers that retained the parent pollinia on the column and did not have naturally deposited pollinia on the stigma were used for artificial pollination. For each orchid, the ripe pollinia were removed with a sterile toothpick and smeared on to the ripe stigma of another predetermined individual after its ripe pollinia had been removed. Each artificially pollinated orchid flower was covered with a small khaki cotton bag, closed at the bottom with an in-built cotton thread tie, and tied around a wooden skewer to support the capsule weight in order to prevent grazing, seed loss at capsule dehiscence and interference from the natural pollinator.

Capsules were collected when ripe (brown) on October 30, 2000 and on November 12 and 16, 2001. The stalk was cut below the bag and the capsules were transported back to the laboratory still enclosed inside the bags to avoid seed loss from split capsules. Once bags had been opened in the laboratory, capsules and seeds were stored in individually labeled paper envelopes over dry silica gel in a sealed container at 4°C.

Seeds were assayed for viability using the fluorescein diacetate (FDA) method of Pritchard (1985) but without surface-sterilization. Replicate samples of more than 50 seeds per capsule were tested between 11 and 43 days after collection. Seeds with the embryo completely stained (fluorescent) were considered viable. Minitab was used to analyze the effect of the pollen and ovule parent types on seed viability against a null hypothesis of no difference, using a non-parametric test (Kruskal–Wallis), since the data could not be normalized. Seeds from grazed, lost or moldy capsules were neither counted nor included in the statistical analysis.

### UV Reflectance and Spectrophotometry

Hymenopterans are attracted by ultraviolet and yellow but also by high color contrast in the flowers and between the flowers and the background (Streinzer et al., 2010; Rakosy et al., 2012; Gaskett et al., 2017). The probability of accurate discrimination by a hymenopteran trichromat can be accurately modeled from the honeybee by determining the Euclidean distance between the respective loci in the color space (von Helversen, 1972a; Peitsch et al., 1992; Briscoe and Chittka, 2001; Gaskett and Herberstein, 2010; Garcia et al., 2017, 2020).

Consistent with permits, in 2001 eight flowers representing each flower category and some subtypes were cut at the stalk base, placed in test-tubes of water and immediately transported back to the laboratory on ice for spectral measurement. Hymenopteran pollinators have vision sensitive to UV radiation, and parts of flowers frequently have UV reflecting signals not normally visible to the human eye (Kevan et al., 2001). The reflectance spectra of sepals and labella were measured using a Varian DMS100 double beam UV-visible spectrophotometer fitted with a diffuse reflectance attachment at 10 nm intervals from 300 to 650 nm relative to a Varian pressed polytetrafluoroethylene powder standard.

To determine if flower color phenotypes were sufficiently spectrally variable as to be discriminated by the likely thynnine wasp pollinator, the spectral data were modeled in the Hexagon color space (Chittka, 1992), assuming typical illumination of a midday open sky equivalent to CIE D6500 (Judd et al., 1964; Wyszecki and Stiles, 1982) expressed as photon flux (Spaethe et al., 2001). We used the spectral sensitivity functions for the three different photoreceptors present in the honeybee (*Apis mellifera*) reported by Peitsch et al. (1992) assuming as adaptation background a 10% reflectance achromatic background (Dyer, 1998).

The probability of accurate discrimination of colors can be predicted from the geometric distance between respective flower colors in a given color space through a discrimination function that incorporates the psychophysics of how color differences are perceived by an animal (von Helversen, 1972a,b). Such a function has been determined for free flying honeybees from experimental data on color discrimination under absolute conditioning (Garcia et al., 2018, 2020), which we used with Euclidean distance in the Hexagon color space as the predictor (independent) variable. Specifically if the probability of discrimination exceeds 70% (von Helversen, 1972a,b) for respective flowers, or flower parts, such colors are above a discriminable threshold for a hymenopteran pollinator. Our analyses thus considered (i) if different flowers had colors that were above or below threshold and (ii) if the colors of the sepals and labella of the same plant were above or below threshold.

## Results

### Sample Population – Tagged and Mapped Orchids

Eighty-one flowering orchids were first categorized and tagged in 2000, 49 in 2001, 70 in 2003, and 64 in 2004 (total of 264). Three orchids in 2000 could not be categorized; one had flowers with calli extending onto its tepals and the other two presented various abnormalities.

The mean number of categorized and tagged plants that flowered per year in 2000, 2003, and 2004 was 71 – 7 (excluding 2001 data because of bias). On average, there were 21% Category 1, 26% Category 2, 30% Category 3, 15% Category 4, and 8% Category 5 flowers (Table 2). The proportions of categories in tagged orchids were unequal in each year and in total (χ^2^ = 36.1, *p* < 0.001) with Categories 1–3 together comprising 77% of the total. The proportions were biased to less common types in 2001 (χ^2^ = 5.6, *p* = 0.232) because only those were still required for the artificial pollination matrix and so the 2001 data were excluded from all except artificial pollination analysis. The proportions of the categories did not differ significantly between 2000 and 2003 (χ^2^ = 7.8, *p* = 0.098) or 2003 and 2004 (χ^2^ = 4.460, *p* = 0.347) but did differ between 2000 and 2004 (χ^2^ = 19.346, *p* = 0.001) and overall (χ^2^ = 22.0, *p* = 0.005). The main difference was the greater proportion of Category 3 and lesser proportion of Category 4 between 2000 and 2003–2004.

**FIGURE 2:**
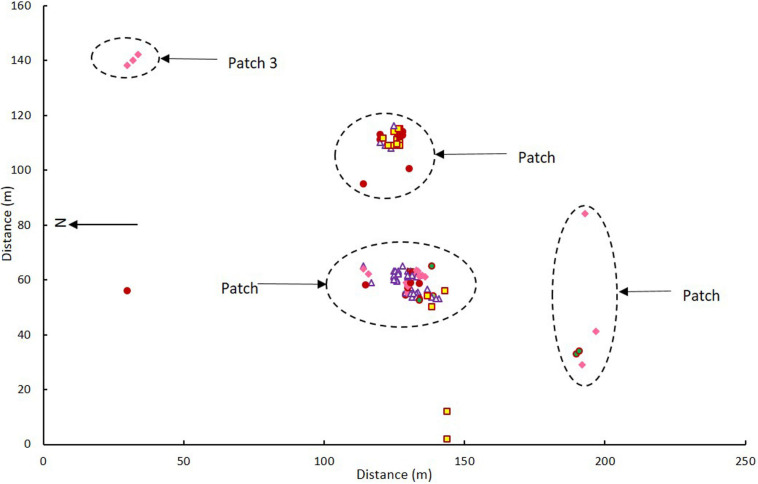
Map of *Caladenia* categories at Deep Lead in patches 1–4 in 2000–2001. Key to flower types: Category 1 solid maroon circles, Category 2 white triangles with maroon borders, Category 3 yellow squares with maroon borders, Category 4 solid pink diamonds, Category 5 green circles with maroon borders.

The flowering orchids grew in two adjacent larger patches with smaller numbers of more scattered individuals in three smaller patches elsewhere on the site (Figure 2). There were no obvious differences in categories among patches (χ^2^ = 7.032, *p* = 0.134). Note that these were flowering orchids, not the entire population, as plants were only tagged on 1–2 days in mid-flowering season each year and there were also many plants without flowers.

### Molecular Analysis

#### Sequencing

**FIGURE 3:**
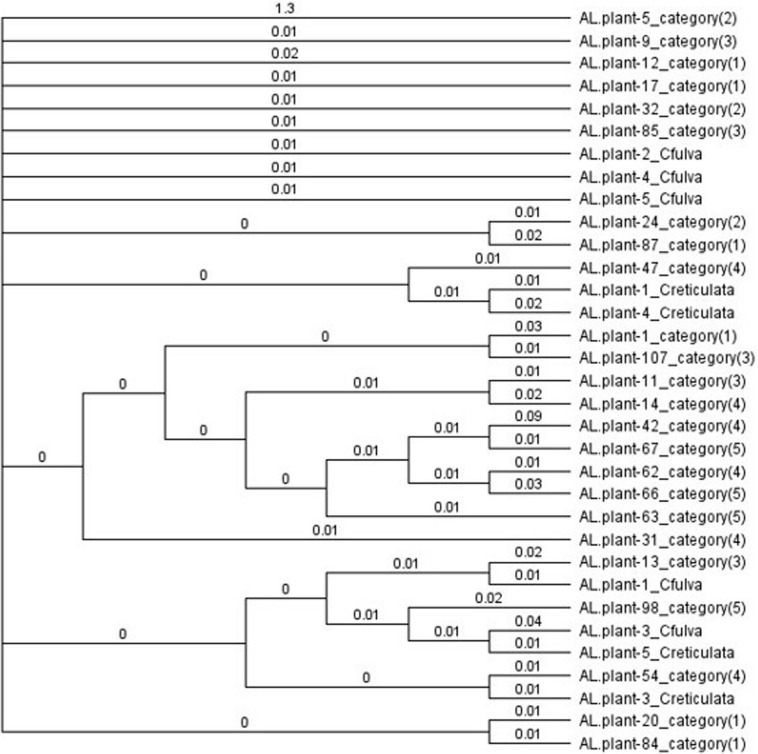
Bayesian cladogram from concatenated ITS, trnT-F, and matK sequences, showing comparative relationships and intermixing of flower color categories and authentic samples of *Caladenia fulva* and *C. reticulata* collected in the same conservation reserve. Genetic distances derived from the posterior output are shown next to the branches.

Of the 52 DNA samples tested, 48 produced a single ITS product, 49 a single trnT-trnL product, all 52 a single trnL intron product, 51 a single trnL-trnF product and 37 a single matK2 product and so were sequenced. Some sequences (5 ITS, 6 trnT-trnF, and 15 matK2) were of poor quality and were omitted from the final analysis. Alignment and concatenation of the remaining 33 sequences (which included at least three sequences from each of the five categories and authentic species) showed that sequence homologies varied from 96.3 to 97.9% except for two sequences (for plants 42 and 5). The phylogenetic tree formed one large clade containing all but these two sequences (Figure 3). Phenotypes and authentic species were mixed throughout rather than separating into categories or authentic species. This mixing also occurred when Maximum Likelihood was used to analyze sequences of each region separately (Supplementary Figures 1–5) or the concatenated sequences (Supplementary Figure 6). In MEGA7, calculated values for mean distance (*d*) were less for the nuclear ITS sequences (0.001–0.025) than for chloroplast sequences (0.000–0.114). For ITS sequences, mean diversities within and among categories (including *C. fulva* and *C. reticulata* samples) were all small (*d* = 0.000–0.001) and the coefficient of differentiation was 0.155 – 0.120. For chloroplast sequences, mean diversities were also small (0.001–0.006) and coefficients of differentiation were −0.006 – 0.012 to 0.082 – 0.036.

#### ISSR

**FIGURE 4:**
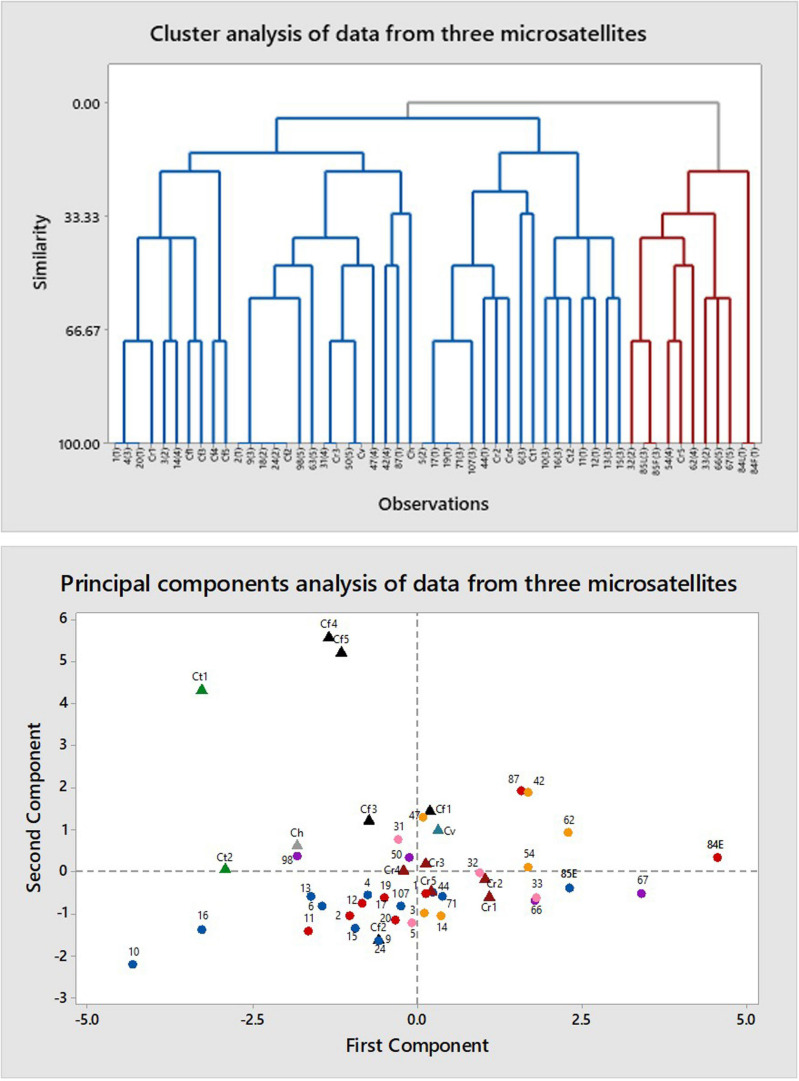
Multivariate analysis of data from amplification of DNA of *Caladenia* species from Deep Lead and others by primers for three microsatellites: (GACA)_4_, (CAT)_5_, and (GTG)_5_. Key to symbols: triangles, authentic species (black, *Caladenia fulva*; maroon, *C. reticulata*; gray, *Caladenia hastata*; teal, *Caladenia venusta*; green, *C tentaculata*); circles, specimens of polymorphic forms (red, Category 1; pink, Category 2; blue, Category 3; orange, Category 4; purple, Category 5).

The primers (CAT)_5_, (GTG)_5_, and (GACA)_4_ all produced multiple bands. Cluster analysis resulted in five large clusters, each containing more than one phenotype and one or more authentic samples (Figure 4). Principal components analysis grouped all phenotypes together in one large cluster without separating by phenotype. The samples of authentic species were scattered among the category samples except for two samples of *C. fulva* and one of *C. tentaculata*. Heterozygosity values were N_*e*_ = 1.258 – 0.028 and unbiased H_*e*_ = 0.157 – 0.016 and Shannon’s Information Index (I) was 0.242 – 0.022. AMOVA resulted in φ_*PT*_ = 0.085 (*p* = 0.010) for categorical samples alone, with 91% of variation within categories and only 9% among categories. Corresponding values including authentic *C. fulva* and *C. reticulata* were 0.130 (*p* = 0.001) with 87% of variation within categories and only 13% among categories.

### Natural Pollination

A total of 134 plants was monitored for natural pollination (70 in 2003 and 64 in 2004) (Table 3). Only 21 (15.7%) produced a capsule with viable seeds; this varied from 10/70 (14%) in 2003 to 11/64 (17%) in 2004.

**TABLE 3:** Variation in flowering and natural pollination with category and patch in which they grew in *Caladenia* species at Deep Lead in 2003 and 2004.

**Criterion**	**2003**		**2004**		**2003–2004**		
	**No. flowers**	**No. fruit**	**No. flowers**	**No. fruit**	**No. flowers**	**No. fruit**	**% success**
**Category**							
1	17	3	13	1	30	4	13.34.8
2	18	4	12	2	30	6	20.04.6
3	23	2	27	7	50	9	18.011.9
4	8	1	4	0	12	1	8.32.4
5	4	0	8	1	12	1	8.32.4
Total	70	10	64	11	134	21	15.7
**Patch**							
1	15	5	20	6	35	11	31.71.7
2	14	3	10	3	24	6	25.74.3
3	7	1	3	0	10	1	7.171
4	8	0	8	1	16	1	6.36.3
5	26	1	23	1	49	2	4.10.3
Total	70	10	64	11	134	21	15.7

*Overall 15.7% success (21/134).*

**FIGURE 5:**
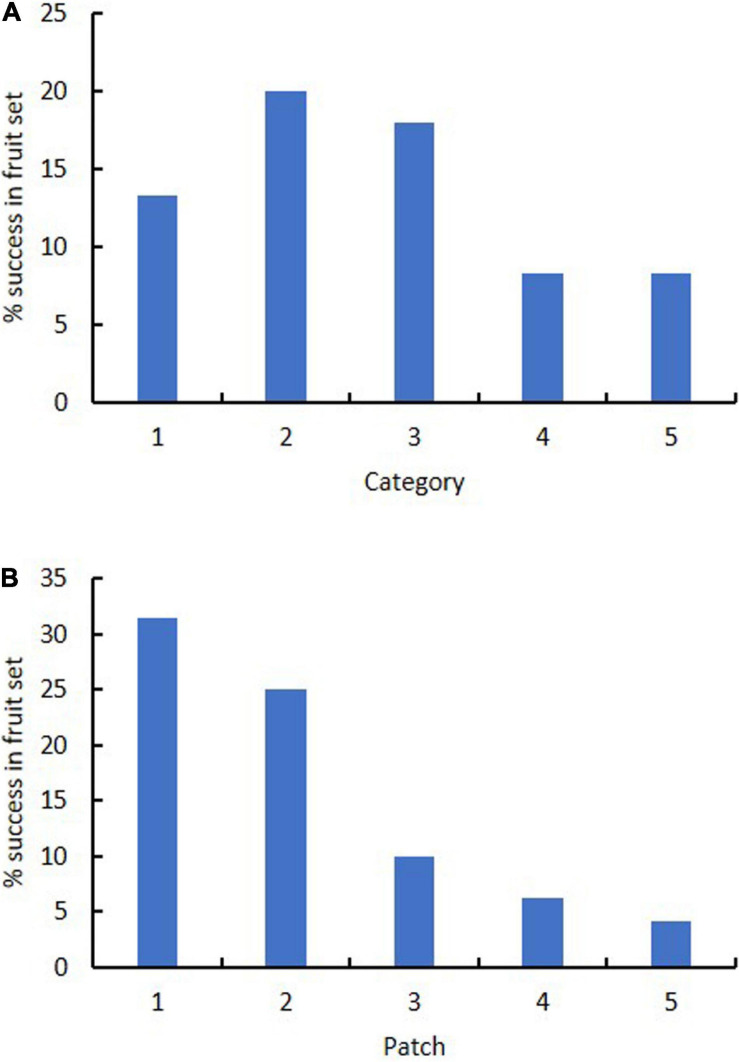
Effect of **(A)** flower color category (1–5) and **(B)** patch (1–5) on percentage success in fruit set in *Caladenia* species at Deep Lead in 2003–2004.

Category of flower color did not affect success in fruiting. Success in fruiting following natural pollination ranged from 8% (Categories 4–5) to 13–20% (Categories 1–3) (Table 3 and Figure 5). Plants in Categories 1–3 (those with 82% of the flowers) produced 91% of the fruits (Category 3 alone produced 43%). However, category itself had no significant effect on relative success in fruiting (χ^2^ = 8.6, *p* = 0.072). There was also no significant deviation from expected numbers of fruit and flower numbers within Categories 1–3 (χ^2^ = 0.358, *p* = 0.836). Alternative measures of association for data from all categories together also all had values close to zero (no association with category): Cramer’s V-square = 0.008, Pearson’s *r* = −0.035, Spearman σ = −0.030, Goodman–Kruskal’s τ = 0.008, Kendall’s τ-b = 0.027. By contrast, linear regression showed that the number of fruits was significantly affected by the number of flowers when data were arranged by category (fruit number = 0.2119 (flower number) – 1.4783, *R*^2^ = 0.9562).

Patch strongly affected success in fruiting. Success in fruiting following natural pollination ranged from 1 to 10% for patches 3–5 to 25–31% for patches 1–2 (Table 3 and Figure 5). Plants in patches 1–2 (with 44% of the flowers) produced 81% of the fruits on site. Patch number had a significant effect on flower number (χ^2^ = 38.3, *p* < 0.001), fruit number (χ^2^ = 17.8, *p* < 0.001) and % fruiting success (χ^2^ = 38.3, *p* < 0.001). Linear regression showed no significant relationship of fruit number with flower number arranged by patch [fruit number = 0.0933 (flower number) + 1.6996, *R*^2^ = 0.1125]. There was also other evidence of association of patch number with fruit number per patch, as values were all positive and deviated from zero: Cramer’s V-square = 0.116, Pearson’s *r* = 0.292, Spearman’s σ = 0.314, Goodman–Kruskal’s τ = 0.116, Kendall’s τ-b = 0.284.

### Artificial Pollination

Seventy of the 91 crosses (77%) in 2000–2001 resulted in the formation of capsules with seeds that could be tested for viability (Table 4 and Supplementary Table 1). Of the remaining 21 artificially pollinated plants, seven did not form a capsule, eight were grazed, five had capsules that were moldy, and one plant could not be re-located. Capsule formation (not including grazed, lost or moldy capsules) was greater than 70% for all combinations, except for one (between Categories 3 and 5), in which only one capsule was produced from three crosses. Every combination except one (Category 3 with Category 5) produced a capsule with reciprocal crosses. Although crossing of Category 5 pollinia to Category 3 stigmas produced a capsule, the reciprocal cross (Category 3 pollinia to Category 5 stigmas) did not produce a capsule.

**TABLE 4:** Capsule formation (% and number excluding moldy ones) and seed viability (%) of *Caladenia* categories from *in situ* artificial pollination experiments.

**Criteria**	**Capsule formation (%) No. capsules formed (n)**					**Seed viability (%) (mean ± SE)**				
**Category**	**1**	**2**	**3**	**4**	**5**	**1**	**2**	**3**	**4**	**5**
Category 1	100	71	100	100	100	60 ± 1	53 ± 17	82 ± 3	60 ± 3	54 ± 7
	*n* = 7	*n* = 7	*n* = 6	*n* = 7	*n* = 2					
Category 2		100	100	100	100		60 ± 13	51 ± 14	55 ± 10	45 ± 15
		*n* = 6	*n* = 8	*n* = 6	*n* = 7					
Category 3			86	83	33			45 ± 14	30 ± 14	20 ± 20
			*n* = 7	*n* = 6	*n* = 3					
Category 4				100	100				19 ± 19	79 ± 15
				*n* = 2	*n* = 2					
Category 5					100					89
					*n* = 1					

Seed viability by the FDA test varied from 0 to 97% with an average of 53% (Table 4 and Supplementary Table 1). There was no significant difference in viability with either pollen parent category (Kruskal–Wallis, *H* = 3.59, *p* = 0.464) or ovular parent category (Kruskal–Wallis, *H* = 6.16, *p* = 0.188). Seed viability in naturally pollinated plants in 2000 was 0–93% (mean 61 – 3%) in capsules from plants of Categories 1–4 (Category 5 plants had no capsules from naturally pollinated plants).

### UV Reflectance and Spectrophotometry

**FIGURE 6:**
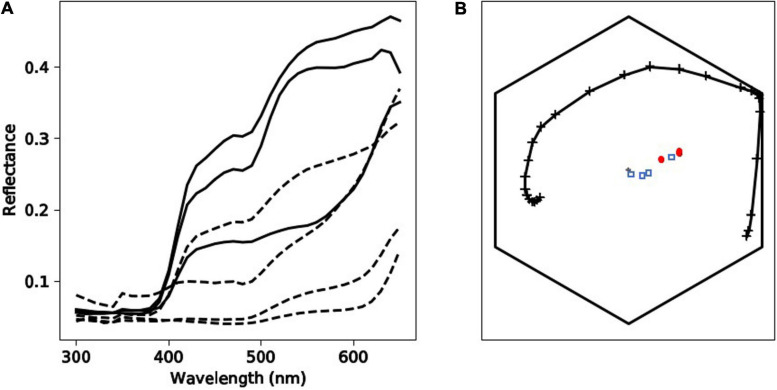
Spectral qualities of *Caladenia* flowers from Deep Lead. Panel **(A)** depicts spectral profiles of tepal (solid black lines) and labellum (dashed black lines) regions of flowers from four different categories (1–4) of the orchid *Caladenia fulva*. Panel **(B)** shows the position of loci corresponding to the samples of *C. fulva* in the hexagon color model. In this panel, marker types identify the different flower regions: tepals (red circle markers) or labella (blue square markers). The spectral loci for *Apis mellifera* as a model of a trichromatic hymenopteran pollinator in the Hexagon color space is indicated by the solid, black line with cross markers.

Tepal reflectances showed an increase in reflection at about 400 nm (Figure 6), which is typical of hymenopteran-pollinated UV-absorbing white flowers around the world, including Australia (Kevan et al., 1996; Dyer et al., 2012; Bischoff et al., 2013).

To understand if the categorization of flower colors (Table 1 and Figure 1) is relevant to the color visual system of hymenopteran trichromats it is important to consider two main scenarios including (i) whether different flowers had colors that were above or below the color threshold perceivable by a pollinator and (ii) if the sepals and labella of the same plant can be discriminated as from each other by a pollinator.

In scenario (i) and then separately comparing variance between the tepals of different plants, in 67% of cases these colors were above discrimination threshold, and there was also variance above threshold in 67% cases for the labella (Table 5). The evidence that flower color is variable in pollinator perception is confirmed by scenario (ii) where color within individual flowers comparing sepals and labella is above threshold in 67% of cases. Finally, comparing remaining possibilities (e.g., sepals versus labella comparisons of different flowers) then in 78% of cases flowers had coloration above discrimination threshold (Table 6). These results show that the color variation used to classify flowers by human observers is often perceivable by hymenopteran pollinators.

**TABLE 5:** Color and green contrast values for the measured sepals (*n* = 3) and labella (*n* = 4) of *Caladenia* species from Deep Lead.

**Stimulus**	**Category**	**Green contrast**	**Color contrast**
Orchid 1 sepal	1	0.267	0.345
Orchid 1 labellum	1	0.087	0.097
Orchid 2 labellum	2	0.091	0.133
Orchid 5 sepal	3	0.285	0.353
Orchid 5 labellum	3	0.188	0.294
Orchid 6 sepal	4	0.130	0.225
Orchid 6 labellum	4	0.163	0.033

**TABLE 6:** Lower triangular matrix containing Euclidean distances between each possible pair of orchid samples (black normal font) and their corresponding probability of discrimination (blue bold italic font) as predicted by the color discrimination function for *Apis mellifera* when considering absolute conditioning.

	**Category**	**O1 sep.**	**O1 lab.**	**O6 sep.**	**O6 lab.**	**O5 sep.**	**O5 lab.**	**O2 lab.**
O1 sep.	1	0						
	*1*	*0.5*						
O1 lab.	1	0.274	0					
	*1*	*0.842*	*0.5*					
O6 sep.	3	0.130	0.157	0				
	*3*	***0.840***	***0.842***	*0.5*				
O6 lab.	3	0.333	0.066	0.208	0			
	*3*	***0.842***	*0.505*	***0.842***	*0.5*			
O5 sep	4	0.011	0.284	0.137	0.341	0		
	*4*	*0.5*	*0.842*	*0.841*	*0.842*	*0.5*		
O5 lab.	4	0.056	0.222	0.074	0.278	0.063	0	
	*4*	*0.5*	***0.842***	*0.570*	*0.842*	*0.5*	*0.5*	
O2 lab.	2	0.238	0.036	0.121	0.097	0.248	0.185	0
	*2*	***0.842***	*0.5*	***0.837***	*0.781*	***0.842***	***0.842***	*0.5*

*A probability of discrimination equal to 0.5 indicates that choices are done at random, i.e., a bee is unable to discriminate a difference in color. Stimuli names are abbreviated from those in Table 3: orchid (O), sepal (sep.), and labellum (lab.).*

## Discussion

The overarching aim of this study was to assist the recovery plan for conservation of *C. fulva* by deciding if all flower color variants (categories) should be included or excluded in plans for artificial pollination and eventual use of *ex situ* plants raised from such seeds for augmentation of the wild population or re-introduction.

The evidence presented here suggests that all flower color categories should be included in recovery actions for *C. fulva*. Firstly, phylogenetic trees did not separate categories or putative parental species and all measures of diversity were small. Secondly, there were no differences in natural seed set among categories of flower color and so no category was disadvantaged in pollination. Thirdly, there were no post-zygotic barriers to artificial cross-pollination or differences in viability in the seeds produced. Lastly, modeling pollinator color vision both within and between flowers suggested that a hymenopteran pollinator should frequently be able to discriminate amongst categories of flower color; the significance of this is discussed below.

### Conservation

All molecular evidence suggests that all categories and the authentic samples of *C. fulva* at the site belong to one potentially interbreeding population that consists of one molecular operational taxonomic unit (MOTU) (Floyd et al., 2002). Since all patches contained all phenotypes of *C. fulva*, the origin of the flower color variation is likely to be genetic rather than environmental. The scattering among the clades of sequences of individuals collected as *C. reticulata* in the same reserves suggests that they too are not reproductively isolated from *C. fulva*. The descriptions of *C. fulva* and C. *reticulata* overlap, as do their ranges (Jeanes, 2015). Carr (1991) thought that *Caladenia audasii* might be a “derived form of *C. fulva*” and sequencing of the sole plant suspected to be “*C. audasii*” at the site would be worthwhile. Swarts et al. (2014) found that some other species in the *C. reticulata/Caladenia patersonii* complexes in this region of south-eastern Victoria were indistinguishable by similar molecular analyses and shared the same thynnine wasp pollinator. Further investigations similar to those described here using more discriminatory sequences or next-generation sequencing may elucidate the relationships among spider-orchids in the *C. patersonii/C. reticulata* complexes.

The high fruit set (77%) and seed viability (53%) from artificial pollination suggest that there are no post-zygotic barriers to cross-pollination among flower color categories at the site, as is common in orchids (Scopece et al., 2014; Baguette et al., 2020). The average seed viability was similar to that found in other orchids (Pritchard, 1985; Huynh, 1999; Sharma et al., 2000). Artificial pollination normally results in consistently greater fruit set than in natural pollination (Alexandersson and Agren, 1996; Primack and Stacy, 1998). It would be ideal to perform classical Mendelian studies of the flower colors of the progeny but there are currently considerable technical difficulties in raising all progeny to maturity (Wright et al., 2009). Repetitive use of plants to increase population size for conservation purposes must also take into account the cost of reproduction on the individual plant (Snow and Whigham, 1989; Primack and Hall, 1990; Alexandersson and Agren, 1996; Primack and Stacy, 1998; Tull, 1998; Coates et al., 2006; Coates and Duncan, 2009).

### Natural Pollination

Fruit set occurred in only 16% of flowers and thus appears low, but is consistent with fruit set data reported for sexually pollinated rather than food-rewarding or food-deceptive species (Gregg, 1989; Tremblay et al., 2005; Jersáková et al., 2006; Vandewoestijne et al., 2009; Scopece et al., 2010; Swarts et al., 2014; Gaskett, 2011; Stejskal et al., 2015; Baguette et al., 2020), including *Caladenia* (Phillips et al., 2009b). This figure is common among natural populations of orchids with a variety of pollination strategies (Nilsson, 1979; Gill, 1989; Waite et al., 1991; Tull, 1998; Waite and Farrell, 1998; Willems and Melser, 1998) and is unlikely to limit recruitment (Tull, 1998).

Patches 1 and 2 together contributed 81% of the seed on site from only 44% of flowers, as noted in two other orchids overseas (Zimmerman and Aide, 1989; Tull, 1998). These patches were the largest on the site, by contrast with the negative trend in fruit set with flower number in European orchids (Tull, 1998; Baguette et al., 2020). Perhaps only large clusters of *C. fulva* produce enough odor to attract pollinators. Baiting attempts may thus need to include larger numbers of potted plants, as for *Caladenia colorata* (Reiter et al., 2018b). Also patches 1 and 2 are on the top of a small rise and are less shaded and therefore sunnier than other patches. Thynnine wasps are more common in sunny warm spring days (Peakall, 1990; Bower, 1996). So long as the flower color phenotypes in patches 1 and 2 continue to resemble those in the population, this bias is unlikely to change the relative proportions of flower color phenotypes in future.

The spectral profiles of the color signals displayed by *C. fulva* in a model of hymenopteran color vision are consistent with pollination by a hymenopteran insect (Chittka, 1992; Dyer et al., 2012; Garcia et al., 2017, 2018, 2020) and observed color and shape variation can be explained as an adaptation to facilitate pollination by hymenopteran insects in this orchid species. This is critical in understanding why *C. fulva* retains a variety of flower colors and patterns, when the plants with the flowers most attractive to a pollinator should be the most successful in reproduction and the population should gradually drift genetically to favor that type (Schiestl and Johnson, 2013). Two factors may be important here: (1) the ability of hymenopteran pollinators to learn from experience and (2) the likely availability of potential hymenopteran pollinators.

Firstly, hymenopterans such as bees and wasps are capable of learning colors and patterns of rewarding and non-rewarding flowers (e.g., Howard et al., 2019). A rewarding orchid flower that delivers food such as nectar will be remembered and visited in future whereas a non-rewarding one will be avoided in future. Therefore, there is potentially an advantage for non-rewarding orchids in having a variety of flower colors and patterns that encourage multiple hymenopteran visits to flowers with slightly different colors or scents. The lack of reward with more common flower colors means that rarer flower colors are actively sought after and so the rarer categories persist in the population in proportion to their total flower numbers. This has been termed negative frequency-dependent selection (Smithson and Macnair, 1997). The learning abilities of hymenopterans are vital in this strategy (Garcia et al., 2020), as pollinators without such memories are indiscriminate in the search for the next flower.

Such signal variability has previously been shown to promote fitness benefits for orchids with non-rewarding flowers (Wong and Schiestl, 2002; Wong et al., 2004; Gaskett, 2011; Dyer et al., 2019; Jiménez-López et al., 2020). For example, sexually deceptive orchid *Ophrys* species display different spatial patterns in conspecific flowers (Stejskal et al., 2015; Paulus, 2019). This avoids pollinators habituating to non-rewarding signals and so increases the probability of multiple flower visits by an individual pollinator (Schiestl, 2005; Jersáková et al., 2006; Delle-Vedove et al., 2017; Dyer et al., 2019). Field experiments suggest that bees prefer visiting similar but discriminable flowers rather than distinctly different colors, a perceptual magnet-type effect that may benefit rarer colored flowers in non-rewarding flower species (Peter and Johnson, 2008; Dyer and Murphy, 2009). Thus species with non-rewarding flowers appear to benefit from having dissimilar colors (Gaskett, 2011; Rakosy et al., 2012) and scents (Delle-Vedove et al., 2017) that potentially confuse the decision-making of pollinators and encourage outcrossing in the orchid (Streinzer et al., 2010; Paulus, 2019). Our observations from *C. fulva* using several lines of inquiry are consistent with this model, and explain the presence of the color variations previously reported for human observers.

Secondly, the availability of a reliable pollinator would be expected to drive plants with a variety of flower colors to evolve in the direction of the flower color most attractive to a reliable pollinator. Different flower colors may appeal to different potential pollinators in the plants’ habitat and so variety in flower color (purple or yellow) may result in pollinator specialization, as in the orchid *D. sambucina* (Gigord et al., 2001). It may lead even further, into speciation, as in the sexually deceptive orchid genus *Ophrys* in Europe (Baguette et al., 2020) and *Drakaea* in Australia (Gaskett et al., 2017). In *Ophrys*, directional selection in favor of attractiveness to specific hymenopteran pollinators is thought to have led to the scent and appearance of the labellum in the small flowers being gradually modified to resemble a female bee that is attractive to a flying male bee seeking a mate, e.g., *Drakaea* (Gaskett et al., 2017), *Ophrys* (Baguette et al., 2020). This strategy works well when potential pollinators are abundant in highly conserved habitats.

However, this strategy works poorly when potential hymenopteran pollinators are sparse and thus unreliable in highly disturbed habitats where orchids and their pollinators are few. In this case, as in *C. fulva* in the Victorian goldfields, there is insufficient selection pressure toward modification of the labellum or tepals to resemble a female hymenopteran. Food-rewarding orchids are believed to precede both food-deceptive and sexually deceptive species (Weston et al., 2014). Therefore, there are advantages in maintaining the attractive bright colors of ancestral rewarding species and possibly obtaining pollination by suitably sized foraging hymenopterans or other flying insects. For example, red and pink colors increased pollinator attraction in *Ophrys heldreichii* (Streinzer et al., 2009; Spaethe et al., 2010). Also, the early stages in biochemical pathways for color and scent molecules are shared and so changes in color may be linked to differences in scent (Zuker et al., 2002; Knudsen et al., 2006; Majetic et al., 2007; Dormont et al., 2014, 2019).

A further point is that few alternative food-rewarding plants are available to foraging invertebrates in the sparse dry undergrowth. Even small quantities of nectar may constitute an important food source. Evidence that many orchids are truly rewardless is scarce and requires further research (Shrestha et al., 2020). The observation of a *Diamma* species licking the bases of the calli on the labellum (Kuiter, 2016) suggests that nectar may be produced directly on to the labellum, as in *Caladenia concolor* (Reiter et al., 2018b, 2019) and *C. nobilis* (Phillips et al., 2020). Given that encounters with foraging flying insects may be simply by chance, there would be advantages to maintaining a variety of attractive colors. Thus in both cases there are potential advantages for *C. fulva* in maintaining flower color diversity.

Clearly identification of the pollinator(s), analysis of the scents of the different color categories and examination of nectar secretion are needed to resolve these questions. Some orchids are pollinated by bees during the day as well as by moths at night (Delle-Vedove et al., 2017) and so diurnal observations should be included in attempts to find pollinators. Schiestl and Schluter (2009) have also suggested that, following scent, size is more important in pollinator attraction than flower color. *C. fulva* flowers are described as varying greatly in tepal size (50–80 mm long × 5–8 mm wide) (Jeanes, 2015). Extreme differences in the size of flowers were noted on site only in Category 5 but measuring flowers and their relative success in fruiting may reveal critical dimensions important for pollination.

The yellow-red flower color polymorphism in *C. fulva* is closest to type D of Dormont et al. (2019). As all plants produce deep red color in some parts of the flower, logically all the plants in the population at the site must possess all the required enzymes of the flavonoid synthesis pathway. This pathway is well characterized and is controlled by regulatory genes, resulting in white, yellow, deep red, and purple pigments, depending on the activities of different regulatory genes (Mol et al., 1998; Martens et al., 2003; Narbona et al., 2018; Jiménez-López et al., 2020). Baguette et al. (2020) recently suggested such epigenetic variation to be key to rapid speciation in *Ophrys* species and pointed out that this would explain the lack of genetic variation found by DNA sequencing despite large differences in odor compounds and color patterns among *Ophrys* species, which is also true for *Caladenia* species (Swarts et al., 2014). Further investigation could test for differences in the flavonoid enzymes and their regulatory enzymes among flower color categories.

### Conservation Recommendations

*Caladenia fulva* is typical of polymorphic endangered orchids in that it has a small spatial range and its taxonomic limits are unclear, as in several such orchids worldwide, notably *Ophrys* species in Europe (Vereecken et al., 2010; Schatz et al., 2014; Joffard et al., 2019; Baguette et al., 2020). The results of this study suggest that *C. fulva* should be regarded as one interbreeding species that is polymorphic in flower color (Huxley, 1955; Narbona et al., 2018; Jiménez-López et al., 2020). Flower color polymorphism may be important in enticing a variety of hymenopterans to visit multiple flowers of *C. fulva* and thus enhance natural pollination and outcrossing even when pollinators are scarce.

This study has also resulted in the following recommendations, which are applicable to other polymorphic endangered orchids in which key facts are unknown. For the purpose of establishing an *ex situ* collection for conservation and possible augmentation or re-introduction, the safest option would be only to use seeds from naturally pollinated fruit of different flower colors. If artificially pollinated, all flower colors should be included (in the proportions found on site) to minimize interference with any natural genetic drift in flower color. Variation in numbers and proportions of flower color categories and their fruit set should be monitored regularly to detect any possible natural drift, e.g., increase in frequency of Category 3. Sustained effort should be devoted to finding the natural pollinator(s) and essential requirements, including adequate abundance of food species for the pollinator(s) in the very sparse understory, in order to conserve rather than preserve *C. fulva*.

## Data Availability Statement

Sequences for *C. fulva* have been deposited in GenBank as Accession Numbers MT894435-MT894470 (ITS), MT914511-MT914554 (trnT-trnL), MT914555-MT914601 (trnL intron), MT950637-MT950682 (trnL-trnF), and MT966280-MT966314 (matK2).

## Author Contributions

RR initiated the study. GB and AL conducted the field and molecular research. AD and JG analyzed UV photography and flower color signals. All authors contributed to and agreed with the final manuscript.

## Conflict of Interest

The authors declare that the research was conducted in the absence of any commercial or financial relationships that could be construed as a potential conflict of interest.

## Footnotes

1 https://www.iucn.org/

2 https://bie.ala.org.au

3 www.monash.edu/researchinfrastructure/micromon

4 http://www.megasoftware.net/mega.php

5 http://www.ncbi.nlm.nih.gov/

6 www.geneious.com

7 minitab.com.au

## Supplementary Material

Supplementary Figure 1 Phylogenetic relationships of *Caladenia* species with polymorphic flower color collected at Deep Lead as shown by nuclear ITS sequences. The evolutionary history was inferred by using the Maximum Likelihood method based on the Tamura-Nei model (Tamura and Nei, 1993). The tree with the highest log likelihood (−1231.23) is shown. The percentage of trees in which the associated taxa clustered together is shown next to the branches. Initial tree(s) for the heuristic search were obtained automatically by applying Neighbor-Join and BioNJ algorithms to a matrix of pairwise distances estimated using the Maximum Composite Likelihood (MCL) approach, and then selecting the topology with superior log likelihood value. The tree is drawn to scale, with branch lengths measured in the number of substitutions per site. The analysis involved 48 nucleotide sequences. All positions containing gaps and missing data were eliminated. There was a total of 608 positions in the final dataset.

Supplementary Figure 2 Phylogenetic relationships of *Caladenia* species with polymorphic flower color collected at Deep Lead as shown by chloroplast trnT-trnL sequences. The evolutionary history was inferred by using the Maximum Likelihood method based on the Tamura-Nei model (Tamura and Nei, 1993). The tree with the highest log likelihood (−1865.73) is shown. The percentage of trees in which the associated taxa clustered together is shown next to the branches. Initial tree(s) for the heuristic search were obtained automatically by applying Neighbor-Join and BioNJ algorithms to a matrix of pairwise distances estimated using the Maximum Composite Likelihood (MCL) approach, and then selecting the topology with superior log likelihood value. The tree is drawn to scale, with branch lengths measured in the number of substitutions per site. The analysis involved 47 nucleotide sequences. All positions containing gaps and missing data were eliminated. There was a total of 546 positions in the final dataset. Evolutionary analyses were conducted in MEGA7 (Kumar et al., 2016).

Supplementary Figure 3 Phylogenetic relationships of *Caladenia* species with polymorphic flower color collected at Deep Lead as shown by chloroplast trnL-trnL sequences. The evolutionary history was inferred by using the Maximum Likelihood method based on the Tamura-Nei model (Tamura and Nei, 1993). The tree with the highest log likelihood (−270.23) is shown. The percentage of trees in which the associated taxa clustered together is shown next to the branches. Initial tree(s) for the heuristic search were obtained automatically by applying Neighbor-Join and BioNJ algorithms to a matrix of pairwise distances estimated using the Maximum Composite Likelihood (MCL) approach, and then selecting the topology with superior log likelihood value. The tree is drawn to scale, with branch lengths measured in the number of substitutions per site. The analysis involved 49 nucleotide sequences. All positions containing gaps and missing data were eliminated. There was a total of 142 positions in the final dataset. Evolutionary analyses were conducted in MEGA7 (Kumar et al., 2016).

Supplementary Figure 4 Phylogenetic relationships of *Caladenia* species with polymorphic flower color collected at Deep Lead as shown by chloroplast trnL-trnF sequences. The evolutionary history was inferred by using the Maximum Likelihood method based on the Tamura-Nei model (Tamura and Nei, 1993). The tree with the highest log likelihood (−6092.21) is shown. The percentage of trees in which the associated taxa clustered together is shown next to the branches. Initial tree(s) for the heuristic search were obtained automatically by applying Neighbor-Join and BioNJ algorithms to a matrix of pairwise distances estimated using the Maximum Composite Likelihood (MCL) approach, and then selecting the topology with superior log likelihood value. The tree is drawn to scale, with branch lengths measured in the number of substitutions per site. The analysis involved 49 nucleotide sequences. All positions containing gaps and missing data were eliminated. There was a total of 519 positions in the final dataset. Evolutionary analyses were conducted in MEGA7 (Kumar et al., 2016).

Supplementary Figure 5 Phylogenetic relationships of *Caladenia* species with polymorphic flower color collected at Deep Lead as shown by chloroplast matK2 (1571f-trnK2r) sequences. The evolutionary history was inferred by using the Maximum Likelihood method based on the Tamura-Nei model (Tamura and Nei, 1993). The tree with the highest log likelihood (−1562.42) is shown. The percentage of trees in which the associated taxa clustered together is shown next to the branches. Initial tree(s) for the heuristic search were obtained automatically by applying Neighbor-Join and BioNJ algorithms to a matrix of pairwise distances estimated using the Maximum Composite Likelihood (MCL) approach, and then selecting the topology with superior log likelihood value. The tree is drawn to scale, with branch lengths measured in the number of substitutions per site. The analysis involved 37 nucleotide sequences. All positions containing gaps and missing data were eliminated. There was a total of 767 positions in the final dataset. Evolutionary analyses were conducted in MEGA7 (Kumar et al., 2016).

Supplementary Figure 6 Phylogenetic relationships of *Caladenia* species with polymorphic flower color collected at Deep Lead as shown by concatenating sequences of nuclear ribosomal DNA (internal transcribed spacer region) with four sequences of chloroplast DNA (three regions in trnT-trnF and matK2 (1571f-trnK2r) region). The evolutionary history was inferred by using the Maximum Likelihood method based on the Tamura-Nei model (Tamura and Nei, 1993). The tree with the highest log likelihood (−12,191.78) is shown. The percentage of trees in which the associated taxa clustered together is shown next to the branches. Initial tree(s) for the heuristic search were obtained automatically by applying Neighbor-Join and BioNJ algorithms to a matrix of pairwise distances estimated using the Maximum Composite Likelihood (MCL) approach, and then selecting the topology with superior log likelihood value. The tree is drawn to scale, with branch lengths measured in the number of substitutions per site. The analysis involved 33 nucleotide sequences. All positions containing gaps and missing data were eliminated. There was a total of 2691 positions in the final dataset. Evolutionary analyses were conducted in MEGA7 (Kumar et al., 2016).
