# Supplementary figures and images for: Why Variation in Flower Color May Help Reproductive Success in the Endangered Australian Orchid Caladenia fulva
Source: Front Plant Sci. 2021 Feb 9;12:599874. doi: 10.3389/fpls.2021.599874 (PMC7899986; doi:10.3389/fpls.2021.599874)

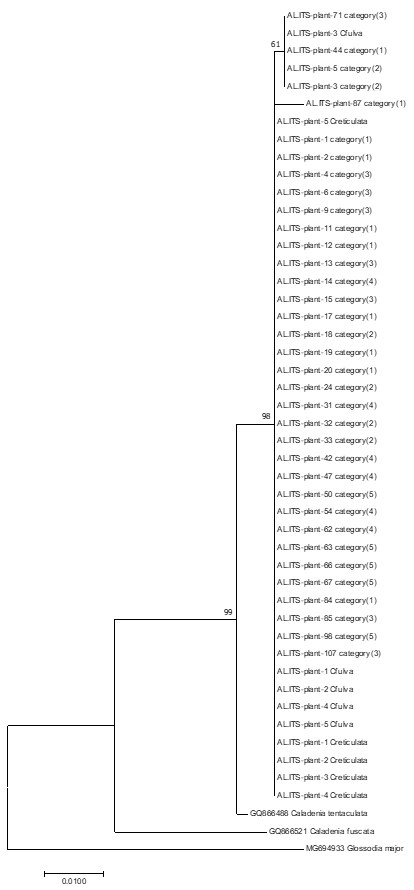

Supplement: Supplementary Figure 1 — Phylogenetic relationships of Caladenia species with polymorphic flower color collected at Deep Lead as shown by nuclear ITS sequences. The evolutionary history was inferred by using the Maximum Likelihood method based on the Tamura-Nei model (Tamura and Nei, 1993). The tree with the highest log likelihood (−1231.23) is shown. The percentage of trees in which the associated taxa clustered together is shown next to the branches. Initial tree(s) for the heuristic search were obtained automatically by applying Neighbor-Join and BioNJ algorithms to a matrix of pairwise distances estimated using the Maximum Composite Likelihood (MCL) approach, and then selecting the topology with superior log likelihood value. The tree is drawn to scale, with branch lengths measured in the number of substitutions per site. The analysis involved 48 nucleotide sequences. All positions containing gaps and missing data were eliminated. There was a total of 608 positions in the final dataset. [file Image_1.JPEG]

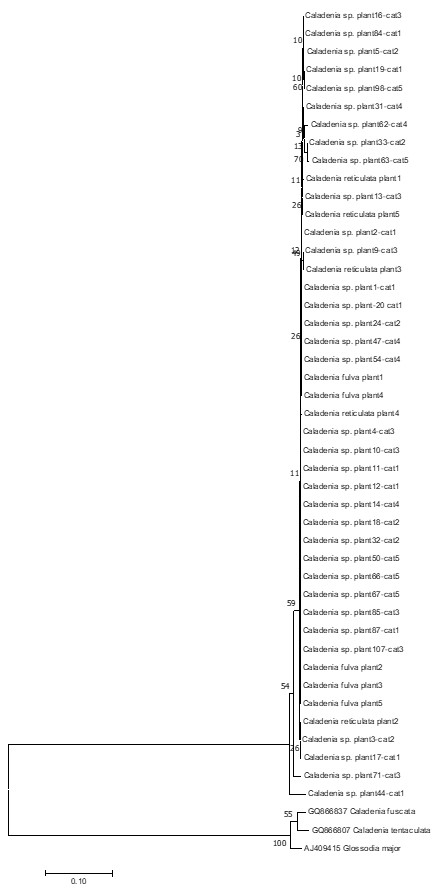

Supplement: Supplementary Figure 2 — Phylogenetic relationships of Caladenia species with polymorphic flower color collected at Deep Lead as shown by chloroplast trnT-trnL sequences. The evolutionary history was inferred by using the Maximum Likelihood method based on the Tamura-Nei model (Tamura and Nei, 1993). The tree with the highest log likelihood (−1865.73) is shown. The percentage of trees in which the associated taxa clustered together is shown next to the branches. Initial tree(s) for the heuristic search were obtained automatically by applying Neighbor-Join and BioNJ algorithms to a matrix of pairwise distances estimated using the Maximum Composite Likelihood (MCL) approach, and then selecting the topology with superior log likelihood value. The tree is drawn to scale, with branch lengths measured in the number of substitutions per site. The analysis involved 47 nucleotide sequences. All positions containing gaps and missing data were eliminated. There was a total of 546 positions in the final dataset. Evolutionary analyses were conducted in MEGA7 (Kumar et al., 2016). [file Image_2.JPEG]

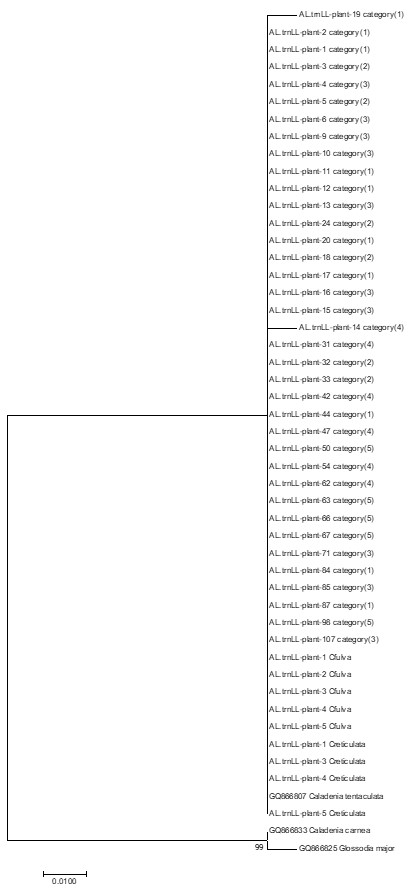

Supplement: Supplementary Figure 3 — Phylogenetic relationships of Caladenia species with polymorphic flower color collected at Deep Lead as shown by chloroplast trnL-trnL sequences. The evolutionary history was inferred by using the Maximum Likelihood method based on the Tamura-Nei model (Tamura and Nei, 1993). The tree with the highest log likelihood (−270.23) is shown. The percentage of trees in which the associated taxa clustered together is shown next to the branches. Initial tree(s) for the heuristic search were obtained automatically by applying Neighbor-Join and BioNJ algorithms to a matrix of pairwise distances estimated using the Maximum Composite Likelihood (MCL) approach, and then selecting the topology with superior log likelihood value. The tree is drawn to scale, with branch lengths measured in the number of substitutions per site. The analysis involved 49 nucleotide sequences. All positions containing gaps and missing data were eliminated. There was a total of 142 positions in the final dataset. Evolutionary analyses were conducted in MEGA7 (Kumar et al., 2016). [file Image_3.JPEG]

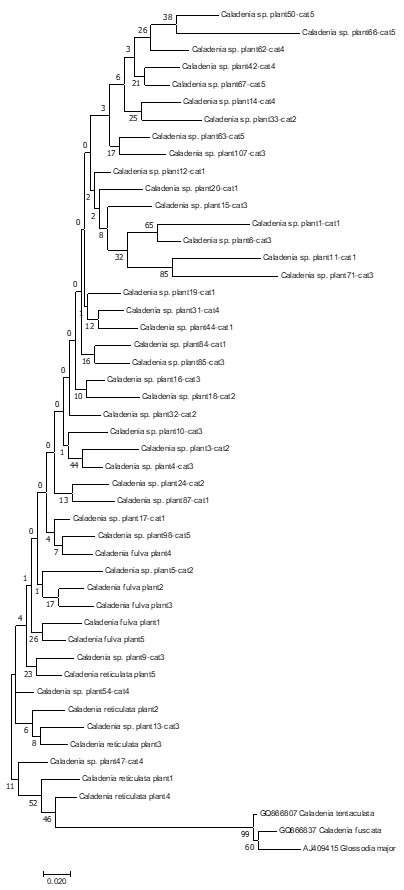

Supplement: Supplementary Figure 4 — Phylogenetic relationships of Caladenia species with polymorphic flower color collected at Deep Lead as shown by chloroplast trnL-trnF sequences. The evolutionary history was inferred by using the Maximum Likelihood method based on the Tamura-Nei model (Tamura and Nei, 1993). The tree with the highest log likelihood (−6092.21) is shown. The percentage of trees in which the associated taxa clustered together is shown next to the branches. Initial tree(s) for the heuristic search were obtained automatically by applying Neighbor-Join and BioNJ algorithms to a matrix of pairwise distances estimated using the Maximum Composite Likelihood (MCL) approach, and then selecting the topology with superior log likelihood value. The tree is drawn to scale, with branch lengths measured in the number of substitutions per site. The analysis involved 49 nucleotide sequences. All positions containing gaps and missing data were eliminated. There was a total of 519 positions in the final dataset. Evolutionary analyses were conducted in MEGA7 (Kumar et al., 2016). [file Image_4.JPEG]

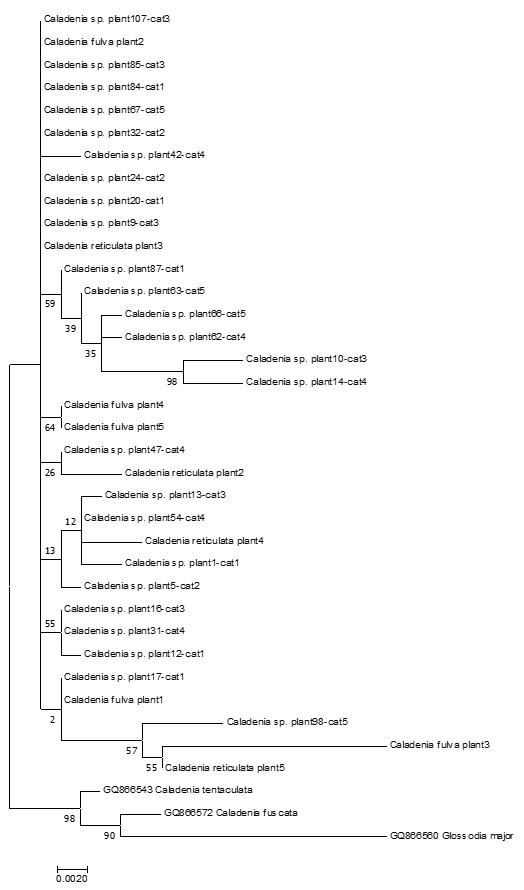

Supplement: Supplementary Figure 5 — Phylogenetic relationships of Caladenia species with polymorphic flower color collected at Deep Lead as shown by chloroplast matK2 (1571f-trnK2r) sequences. The evolutionary history was inferred by using the Maximum Likelihood method based on the Tamura-Nei model (Tamura and Nei, 1993). The tree with the highest log likelihood (−1562.42) is shown. The percentage of trees in which the associated taxa clustered together is shown next to the branches. Initial tree(s) for the heuristic search were obtained automatically by applying Neighbor-Join and BioNJ algorithms to a matrix of pairwise distances estimated using the Maximum Composite Likelihood (MCL) approach, and then selecting the topology with superior log likelihood value. The tree is drawn to scale, with branch lengths measured in the number of substitutions per site. The analysis involved 37 nucleotide sequences. All positions containing gaps and missing data were eliminated. There was a total of 767 positions in the final dataset. Evolutionary analyses were conducted in MEGA7 (Kumar et al., 2016). [file Image_5.JPEG]

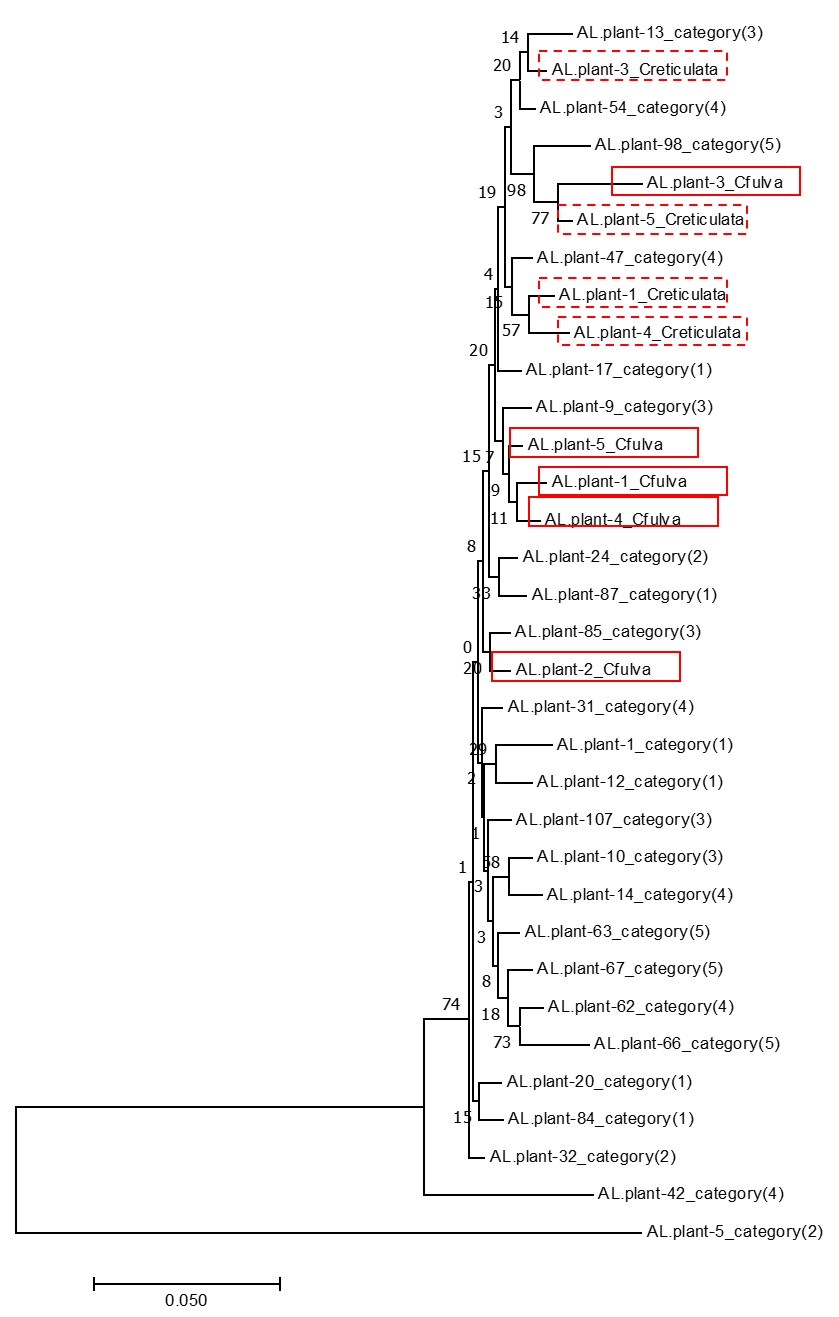

Supplement: Supplementary Figure 6 — Phylogenetic relationships of Caladenia species with polymorphic flower color collected at Deep Lead as shown by concatenating sequences of nuclear ribosomal DNA (internal transcribed spacer region) with four sequences of chloroplast DNA (three regions in trnT-trnF and matK2 (1571f-trnK2r) region). The evolutionary history was inferred by using the Maximum Likelihood method based on the Tamura-Nei model (Tamura and Nei, 1993). The tree with the highest log likelihood (−12,191.78) is shown. The percentage of trees in which the associated taxa clustered together is shown next to the branches. Initial tree(s) for the heuristic search were obtained automatically by applying Neighbor-Join and BioNJ algorithms to a matrix of pairwise distances estimated using the Maximum Composite Likelihood (MCL) approach, and then selecting the topology with superior log likelihood value. The tree is drawn to scale, with branch lengths measured in the number of substitutions per site. The analysis involved 33 nucleotide sequences. All positions containing gaps and missing data were eliminated. There was a total of 2691 positions in the final dataset. Evolutionary analyses were conducted in MEGA7 (Kumar et al., 2016). [file Image_6.JPEG]
