# Supplementary material for: Why Variation in Flower Color May Help Reproductive Success in the Endangered Australian Orchid Caladenia fulva
Source: Front Plant Sci. 2021 Feb 9;12:599874. doi: 10.3389/fpls.2021.599874 (PMC7899986; doi:10.3389/fpls.2021.599874)
Supplement: Supplementary file 7 [file Table_1.DOCX]

**Table S1**. Details of pollination scheme, pollination outcomes and seed viability results.

|  | Category 1 | | | Category 2 | | | Category 3 | | | Category 4 | | | Category 5 | | |
| --- | --- | --- | --- | --- | --- | --- | --- | --- | --- | --- | --- | --- | --- | --- | --- |
|  | Parent | | Seed Viability | Parent | | Seed Viability | Parent | | Seed Viability | Parent | | Seed Viability | Parent | | Seed Viability |
|  | Male | Female | (%) | Male | Female | (%) | Male | Female | (%) | Male | Female | (%) | Male | Female | (%) |
| Category 1 | **002 × 001***  **012 × 020***  **017 × 018***  **018 × 017***  **020 × 012***  **036 × 034***  **082 × 036*** | | **71**  **76**  **0**  **35**  **83**  **84**  **74** | **020 × 022****  **038 × 040***  **040 × 038***  **078 × 082***  090 × 091*  091 × 090*  087 × 086** | | **-**  **61**  **65**  **94**  83  71  - | **010 × 011***  **021 × 081***  **068 × 069***  **069 × 068***  **081 × 021***  084 × 085*  085 × 084# | | **91**  **76**  **73**  **81**  **84**  88  - | **043 × 044***  **044 × 043***  **053 × 054***  **054 × 053***  088 × 089*  089 × 088*  094 × 113*  113 × 094# | | **80**  **85**  **96**  **47**  7  15  91  - | **063 × 065***  **065 × 063***  095 × 098***  098 × 095*** | | **61**  **47**  -  - |
| Category 2 |  | |  | **045 × 049***  **051 × 052***  **052 × 051***  093 × 097*  096 × 099*  097 × 093#  099 × 096* | | **72**  **0**  **97**  61  74  -  58 | **003 × 004#**  **004 × 003***  **005 × 007***  **007 × 005***  092 × 100*  100 × 092*  105 × 101*  107 × 117*  117 × 107* | | **-**  **94**  **44**  **95**  9  79  14  71  1 | **041 × 042***  **047 × 041***  **072 × 062***  **062 × 072#**  106 × 114*  108 × 115?  114 × 106*  115 × 108* | | **61**  **34**  **62**  **-**  40  -  97  38 | **064 × 066a***  **066a × 064***  109 × 116*  112 × 118*  116 × 109*  118 × 112*  120 × 119***  121 × 120* | | **0**  **9**  79  1  77  59  -  87 |
| Category 3 |  | | |  | | | **009 × 083***  **015 × 016***  **016 × 015***  **025 × 026#**  **083 × 009***  110 × 111**  111 × 110*  126 × 127* | | **82**  **0**  **30**  **-**  **63**  -  43  96 | **013 × 058****  **058 × 013****  **060 × 071***  **071 × 060***  127 × 130*  130 × 126* | | **-**  **-**  **0**  **53**  46  78 | **027 × 067*****  **067 × 027***  122 × 123**  123 × 122** | | **-**  **61**  -  - |
| Category 4 |  | | |  | | |  | | | **056 × 057***  **057 × 056***  **073 × 074#**  **080 × 073#**  128 × 129*** | | **38**  **0**  **-**  **-**  - | **050 × 055***  **055 × 050*** | | **94**  **64** |
| Category 5 |  | | |  | | |  | | |  | | | 119 × 121* | | 89 |

**Bold = 2000**; Black = 2001.

*Capsule formation; **No capsule formation; ***Capsule moldy; #Grazed? Lost Tag (115)

Grazed before artificial pollination: 079, 077. #Grazed after artificial pollination (not used in scheme): 010, 024, 025, 029, 075, 076, 080.
